# Anhydrous microwave synthesis as efficient method for obtaining model advanced glycation end-products

**DOI:** 10.3389/fmolb.2024.1484196

**Published:** 2024-11-01

**Authors:** Agnieszka Bronowicka-Szydełko, Katarzyna Madziarska, Aleksandra Kuzan, Łukasz Lewandowski, Joanna Adamiec-Mroczek, Jadwiga Pietkiewicz, Maciej Tota, Maciej Ziomek, Wojciech Stach, Tymoteusz Trocha, Marcin Piersiak, Maciej Pachana, Zuzanna Galińska, Andrzej Korpacki, Olgierd Dróżdż, Janusz Matuszyk, Małgorzata Mitkiewicz, Andrzej Gamian, Kinga Gostomska-Pampuch

**Affiliations:** ^1^ Department of Biochemistry and Immunochemistry, Wroclaw Medical University, Wroclaw, Poland; ^2^ Clinical Department of Diabetology, Hypertension and Internal Disease, Wroclaw Medical University, Wroclaw, Poland; ^3^ Department of Preclinical Sciences, Pharmacology and Medical Diagnostics, Faculty of Medicine, Wroclaw University of Science and Technology, Wroclaw, Poland; ^4^ Clinical Department of Ophthalmology, Wroclaw Medical University, Wroclaw, Poland; ^5^ Faculty of Medicine, Wroclaw Medical University, Wroclaw, Poland; ^6^ Laboratory of Tumor Molecular Immunobiology, Hirszfeld Institute of Immunology and Experimental Therapy, Polish Academy of Sciences, Wroclaw, Poland; ^7^ Laboratory of Medical Microbiology, Hirszfeld Institute of Immunology and Experimental Therapy, Polish Academy of Sciences, Wroclaw, Poland

**Keywords:** advanced glycation end-products, NF-κB, microwave synthesis, pro-inflammatory factors, AGEs, biomarkers

## Abstract

**Introduction:**

Advanced glycation end-products (AGEs) are capable of stimulating oxidative stress and inflammation. This study investigates the synthesis of medium crosslinked AGEs (the most optimal form of AGEs because of soluble in water, used in many assays as markers) and their biochemical properties.

**Methods:**

One of model protein–myoglobin from horse heart muscle (MB) and a chosen respective glycation factor – D-melibiose (mel), acrolein (ACR), D-glucose (glc), 4-hydroksynonenal (4HNE), trans-2-nonenal (T2N), methylglyoxal (MGO) – were subjected to high temperature water synthesis (HTWS) and high temperature microwave synthesis in anhydrous conditions (HTMS). The syntheses were deliberately carried out in two different conditions to check whether adding an additional energy source (microwaves) while lowering the temperature and shortening the reaction time would allow for more effective obtaining of medium-cross-linked AGEs, monitored with SDS-PAGE. Products were analyzed using fluorescence measurements, Enzyme-Linked Immunosorbent Assay (ELISA) and immunoblotting tests and electrophoretic mobility shift assay to evaluate their ability to activate nuclear factor kappa-light-chain-enhancer (NF-κB).

**Results:**

Medium cross-linked AGEs were more efficiently obtained in HTMS. Fluorescence was high for MB-ACR, MB-T2N and MB-glc products. Anti-MAGE antibodies showed reactivity towards MB-mels of HTMS and HTWS, and the MB-4HNEs from HTMS. HTWS products, apart from MB-ACR, did not activate NF-κB, whereas MB-ACR, MB-4HNE, MB-mel, and MB-T2N products of HTMS strongly activated this factor that indicates their strong pro-inflammatory properties.

**Conclusion:**

HTMS is a fast and efficient method of synthesizing medium cross-linked AGEs.

## 1 Introduction

Advanced glycation end-products (AGEs) are products of the non-enzymatic reaction of proteins and reducing sugars that are potentially harmful as capable of stimulating oxidative stress, inflammation, and apoptosis (Bronowicka-Szydełko et al., 2024; Rungratanawanich et al., 2021). They have been the subject of research for more than a century, although first in the context of exogenous production associated with food processing, and only since about 1968 in the context of endogenous production in the body and the clinical consequences of this process (Uceda et al., 2024). This impact is due to at least two facts: proteins modified by glycation lose their original properties, and AGEs themselves can interact with a variety of other proteins, which will also have specific consequences. AGEs bind to a variety of receptors, including receptor for advanced glycation end-products (RAGE), advanced glycation end-product receptor (AGER) and scavenger receptors (Bronowicka-Szydełko et al., 2024; Twarda-Clapa et al., 2022). While the binding of a ligand to AGER or the scavenger receptor allows neutralization of the potentially deleterious effects of glycation by degradation of the product inside the cell which is internalizing the complex (most often a macrophage), the binding of a ligand to RAGE generates a cellular signal to trigger activation of pro-inflammatory mechanisms. Proteins involved here include NADPH oxidase, extracellular signal-regulated kinases 1 and 2 (ERK1/2), mitogen-activated protein kinases (MAPK), phosphoinositide 3-kinase (PI3K), protein kinase B (AKT), and a key effect of this cascade is the production of reactive oxygen species (ROS) and reactive nitrogen species (RNS) (Rungratanawanich et al., 2021) as well as activation of NfκB (nuclear factor kappa-light-chain-enhancer of activated B cells), which triggers the synthesis of numerous pro-inflammatory proteins such as TNF-α and IL-1β (Yue et al., 2022). Hence the more AGEs bind to RAGE, the potentially greater the risk of developing inflammation-based diseases in various tissues, including blood vessels, the gastrointestinal tract, and the nervous system, thus it follows the demonstrated cause-and-effect relationships between the amount of AGEs and diabetes, cardiovascular complications, neurodegenerative diseases, immune diseases, gut microbiome-associated illnesses, and cancers (Rungratanawanich et al., 2021).

The pleiotropic effect of AGE-mediated NF-κB activation is based on activation of a high-molecular-weight, three-subunit IκB (IKK) complex - playing both regulatory (IKKγ domain) and catalytic (IKKα, IKKβ subunits) roles. NF-κB translocates to the nucleus, where it enhances the expression of IL-1β, IL-6, TNF-α and CRP, changes the expression of GLUT-4 in skeletal muscles (Khalid et al., 2022), increases macrophage differentiation towards the pro-inflammatory M1 phenotype (Jin et al., 2015), increases the expression of IFN-γ (Zhang et al., 2019) and further passes the signal through the PKC pathway (Khalid et al., 2022). Due to these metabolical changes, NFκB activation is reported to be associated with: insulin resistance (Assis et al., 2024), lipid metabolism dysregulation (Khalid et al., 2022), hypertension (Peng et al., 2016) and promotion of cardiovascular diseases and diabetic nephropathy (Lal et al., 2002), among others.

The glycation factor that initiated the reaction has a significant effect on the structure and properties of the product. In physiology, these are mostly sugars such as glucose (glc), glucose-6-phosphate and fructose (Twarda-Clapa et al., 2022). Various reactive carbonyls, like glyoxal, methylglyoxal (MGO), glycolaldehyde, glyceraldehyde, diacetyl, and 1- and 3-deoxyglucosone, as well as reactive unsaturated aldehydes such as acrolein (ACR), 4-hydroxynonenal (4HNE) and malondialdehyde also participate in smaller amounts (Alfarhan et al., 2020; Twarda-Clapa et al., 2022). These compounds occur as contaminants in food, water, air–including as components of tobacco smoke, but also endogenously formed during peroxidation of polyunsaturated fatty acids (Alfarhan et al., 2020). A variety of glycation factors are used in laboratory conditions. In the present study, we used the relatively low-reactive but classically physiological D-glucose, but also comparatively ACR, MGO, 4HNE and trans-2-nonenal (T2N).

A rare *in vitro* glycation factor is a disaccharide. The condition for a sugar to initiate glycation is that it must be reducing, so sucrose does not meet this criterion, but already melibiose does. Glycation products derived from the reaction of melibiose (mel) with proteins (mel-derived AGEs, MAGE) were first described more extensively in the literature by Staniszewska et al. (2021). The same team has also developed monoclonal anti-AGEs antibodies that react with antigens in serum and in a variety of tissues (Gostomska-Pampuch et al., 2022a; Indyk et al., 2021; Litwinowicz et al., 2022; Staniszewska et al., 2021; Zubchenko et al., 2022), demonstrating that glycation products analogous to synthetic MAGE realistically participate in the biochemical environment of human tissues, potentially involved in metabolic disorders such as diabetes, atherosclerosis and cancer. While the structure and basic biological properties of the *in vitro* obtained MAGE has been elucidated there is need for structure and origin determination of the natural *in vivo* counterpart, namely, AGE10 (Bronowicka-Szydełko et al., 2021). In the present study, we are verifying the reactivity of anti-MAGE (anti-AGE10) antibodies against other AGEs, for the sake of completing the overview we currently have of glycation products.

Glycation products are formed in reactions involving highly reactive compounds, such as oxoaldehydes or aldoses, with the amino groups of proteins, DNA or lipids. These precursors also contain many other functional groups that are also reactive. Therefore, in *in vitro* syntheses, it is important to select the temperature and time conditions so that the resulting synthesis products are soluble in water (otherwise they could not be used in enzyme immunoassay tests, LC-MS analysis). Our previous studies have shown that the highest percentage of soluble AGEs occurs in mixtures dominated by medium cross-linked AGEs. It was shown, among others, that the largest amount of one of the epitopes of synthetic AGE10, called MAGE (obtained as a result of synthesis carried out in a microwave reactor), was found in medium cross-linked products (Staniszewska et al., 2021). A competitive ELISA test conducted on products obtained from subsequent MAGE chromatographic separation fractions showed that the highest reactivity towards anti-MAGE was exhibited by medium cross-linked compounds (the degree of cross-linking was determined on the basis of the analysis of products separated by electrophoresis using polyacrylamide gels) (Bronowicka-Szydełko et al., 2021). This observation suggested that perhaps adding another energy source (i.e., microwaves), instead of a very high temperature, allows obtaining mixtures of AGEs of various types, in which the medium cross-linked fraction dominates. Therefore, the presented publication compares the efficiency of the reaction carried out at high temperature in an aqueous environment (commonly used to prepare AGEs’ mixtures) with the reaction of the same reagents (model protein with a selected glycating factor) carried out at a lower temperature and shorter time, but in the presence of microwaves (instead of water). Medium cross-linked AGEs are called the products of the reaction of a model protein [e.g., myoglobin (MB)] with a given glycation factor (e.g., reducing sugar or alpha-oxoaldehyde), which, although cross-linked with stable chemical bonds, are still soluble in water. In the presented paper, MB was deliberately used because it is a protein of low molecular weight (17 kDa), thanks to which the confirmation of cross-linking by a given glycation factor is possible using SDS-PAGE electrophoresis. This method enables the detection of glycation products with masses of up to approximately 1,200–1,500 kDa.

The aim of this study was to optimize the conditions for the synthesis of various AGEs to allow obtaining the highest possible share of medium-cross-linked AGEs, and to check the fluorescence ability of received AGEs, along with their immunological properties in reaction with new anti-MAGE antibodies and their pro-inflammatory properties (induction of NF-κB by AGEs). The syntheses used a protein that does not require additional separation from artifacts [which we confirmed using fast protein liquid chromatography (FPLC), the separation results have not been published] and is low molecular mass, which makes it possible to detect medium-cross-linked AGEs using a standard SDS-PAGE electrophoresis. Glycation factors were selected to represent various different groups of compounds that are formed *in vivo* in various disorders of sugar or lipid metabolism (methylglyoxal, trans-2-nonenal, 4-hydroxynonenal) or formed *in vitro* during high-temperature food processing (acrolein–is produced when butter is fried and are delivered to organism with food) and glucose, which is a standard glycation factor. Melibiose were also used as a glycation factor because of capable of obtaining MAGE reactive to anti-MAGE antibodies. Although melibiose is a disaccharide found in honey, in our previous studies we have shown that it allows obtaining an analog of the AGE10 epitope, present in blood serum–a natural analogue of synthetic MAGE product (Bronowicka-Szydełko et al., 2021). In scientific research on the glycation process, synthetic structural analogs of AGEs corresponding to compounds occurring *in vivo* are used. Model AGEs can be applied, among others, to study the structure and properties of the formed compounds, obtain antibodies that recognize specific glycation products, and develop standards and markers used in clinical and diagnostic studies. This paper presents the development of an efficient and rapid method for obtaining basic model AGEs.

## 2 Materials and methods

### 2.1 Synthesis of advanced glycation end-products

All chemicals were from Sigma-Aldrich (Saint Louis, MO, United States), unless otherwise stated. Protein AGEs were generated using either high temperature water synthesis (HTWS) or high temperature microwave synthesis in anhydrous conditions (HTMS) (Staniszewska et al., 2021). In order to obtained glycation products we prepared a miliQ water solution of protein monomer–myoglobin from horse heart muscle (MB) – with a concentration of 300 mg/mL and respective glycation factor (GF): mel–D-melibiose, ACR–acrolein, glc- D-glucose (Serva, Heidelberg, Germany), 4HNE – 4-hydroxynonenal, T2N–trans-2-nonenal, and MGO–methylglyoxal in molar ratio MB/GF of 1/100. The protein solution was pipetted so that the MB content in each synthesis sample was 30 mg. The molar ratio was chosen such that for every amino acid residue that can undergo glycation in protein, there were at least 3 GF molecules, resulting in synthesis of medium cross-linked glycation products (Bronowicka-Szydełko, 2012). Samples for HTMS method were frozen and lyophilized. Next, the MB/GF mixtures were subjected to chosen synthesis method. The HTMS was carried out in the Discover microwave reactor (CEM Corporation, Matthews, NC, United States) in 65°C, 75°C, 85°C, 95°C or 105°C for 5, 10 or 15 min, at a constant power of 200 W. After synthesis the obtained MB-GF products were dissolved in miliQ water. The control for each synthesis was a lyophilized MB/GF sample, not subjected to microwaves. The MB/GF solutions subjected to HTWS method were incubated in the SML 32/250 oven (Zalmed, Warsaw, Poland) in 75°C, 85°C or 95°C for 20, 30, 45, 60 or 120 min. The control for each synthesis was a MB/GF solution, not subjected to temperature. Next, all samples after HTMS and HTWS were sonicated for 15 min in Sonorex ultrasonic bath (Bandelin, Berlin, Germany) and centrifuged for 30 min at 15,000 × g (Sigma Laborzentrifugen GmbH, Osterode am Harz, Germany). The supernatants containing water-soluble AGEs were analyzed using SDS-PAGE.

### 2.2 SDS-PAGE

10 µg of each MB-GF product were diluted 1:1 in Sample Buffer (0.06 M Tris-HCl pH 6.8, 2% SDS, 10% glicerol, 0.025% bromophenol blue, 5% β-mercaptoethanol) and denatured by incubation at 100°C for 5 min. Then, the samples were loaded (10 µg/well) onto a polyacrylamide gel consisting of a 4% stacking and a 12% separation gel. The electrophoretic separation was carried out at a constant current of 40 mA for 90 min in a Mini-Protean Tetra Cell system (Bio-Rad, Hercules, CA, United States). Next, gels was stained with 0.25% Coomassie Brilliant Blue solution (Thermo Fisher Scientific, Waltham, MA, United States) and de-stained with 50 mM methanol, 75 mM acetic acid solution or used for protein transfer. Molecular weights of bands obtained after SDS-PAGE separation were determined using LabImage gel analysis software (Kapelan Bio-Imaging, Leipzig, Germany).

### 2.3 Fluorescence spectra

Verification of the fluorescence properties of MB-GF products obtained in HTMS or HTWS synthesis was necessary to classify them into the appropriate group of AGEs (Birlouez-Aragon et al., 2005; Séro et al., 2013). The measurements were carried out on 0.05 mg/mL aqueous solutions of MB-GF and unmodified MB using a black 96-well plate (100 µL/well) in duplicates. The fluorescence was measured with the following parameters: 1) excitation: 260 nm, emission: 280–430 nm; 2) excitation: 280 nm, emission: 300–450 nm; 3) excitation: 330 nm, emission: 350–500 nm; 4) excitation: 460 nm, emission: 480–600 nm.

### 2.4 Western blotting

For Western blotting analysis, the proteins separated on the polyacrylamide gel were transferred to a PVDF membrane (0.45 µm, Merck-Millipore, Burlington, MA, United States) using a semi-dry Trans-Blot system (Bio-Rad, Hercules, CA, United States) in Tris-glycine buffer [10 mM Tris, 150 mM glycine, 20% (v/v) methanol, pH 8.3]. The transfer was carried out for 2 h at a constant current of 170 mA. Then, the membrane was stained with Ponceau S solution [0.1% (w/v) Ponceau S (Sigma Aldrich, Saint Louis, MO, United States), 5% (v/v) acetic acid] in order to confirm the presence of MB-GF products, and de-stained by washing in miliQ water. Next, the membrane was blocked overnight in a 5% solution of powdered skimmed milk (SM Gostyń, Poland) in TBS-T (15 mM Tris-HCL, 150 mM NaCl, pH 7.5 with 0.05% Tween-20) at 4°C. After washing 3 times in TBS-T, the membrane was incubated for 3.5 h, at 37°C with mouse anti-MAGE monoclonal antibodies (Gostomska-Pampuch et al., 2022b; Staniszewska et al., 2021) diluted 1:2,500 in TBS. Then, the membrane was washed 3 times with TBS-T buffer, followed by incubation for 2 h, at 37°C with goat anti-mouse IgM-HRP secondary antibody (Jacson Immunoresearch, West Grove, PA, United States) diluted 1:5,000 in TBS. The membrane was next washed 5 times with TBS-T and developed with OPD (o-phenylenediamine) solution, according to standard procedure. The results were recorded using a Vilber Lourmat (Collegien, France) detection and analysis system.

### 2.5 Enzyme-linked immunosorbent assay (ELISA)

The 96-well plate was coated with solution of each MB-GF product (1 µg/well) in 100 µL of carbonate buffer (20 mM sodium carbonate, 100 mM sodium bicarbonate, pH 9.6) in order to check the reactivity of obtained HTMS and HTWS products to anti-MAGE antibody. Every sample was applied in 4 replicates. The plate was incubated for 5 h at room temperature. Then the plate was washed three times with TBS-T solution and blocked with 10% skimmed milk powder (SM Gostyń, Poland) solution in TBS-T overnight at 4°C. Next, after washing 3 times with TBS-T, 100 µL/well of antibodies: mouse anti-MAGE monoclonal antibody, diluted 1:4,000 in PBS or rabbit polyclonal anti-MAGE antibodies, diluted 1:100 in PBS (Bronowicka-Szydełko et al., 2021; Staniszewska et al., 2021), were applied on the plate and incubated for 3 h, at room temp. Then, after washing with TBS-T as before, 100 µL/well of secondary antibodies: goat anti-mouse IgM-HRP antibody (Jacson Immunoresearch West Grove, PA, United States) diluted 1:6,000 in PBS or goat anti-rabbit IgG-HRP (Abcam Inc., Cambridge, United Kingdom), diluted 1:4,000 in PBS, were applied. After 2.5 h incubation at room temp, an excess of antibodies was washed out with TBS-T and the reaction was induced with 100 µL/well of developing OPD solution containing citrate buffer, pH 4.5 (0.1 M citric acid, 0.1 M sodium citrate), 1.5 mg/mL OPD, 0.03% (v/v) H_2_O_2_, for 10 min in the dark, at room temp. The reaction was stopped with 40% H_2_SO_4_ (50 µL/well) and the obtained color intensity was measured at 490 nm with a Power Wave XS spectrophotometer (BioTek Instruments, Winooski, VT, United States). The results were normalized by subtracting the absorbance for a negative control where PBS was applied to a well instead of primary antibody.

### 2.6 Cell culture and treatment

Assessment of NF-κB activation by selected MB-GF products was measured in human monocytic cell line (THP-1), obtained from the European Collection of Authenticated Cell Cultures (ECACC). THP-1 cells were propagated in RPMI 1640 medium supplemented with 10% fetal calf serum, 1 mM sodium pyruvate and antibiotics (100 U/mL penicillin G, 0.25 μg/mL amphotericin B, 0.1 mg/mL streptomycin) at 37°C in 5% CO_2_. THP-1 cells were seeded in 24-well cell culture plates at a density of 5 × 105 cells per well (in 1 mL of medium), and then cells were treated for 90 min with 50 μg/mL of following AGEs in duplicates: MB-mel, MB-ACR, MB-4HNE, MB-glc and MB-T2N obtained in HTMS and HTWS methods. In the experiments we used controls in the form of untreated cells (negative control), cells treated with unmodified myoglobin and cells treated with lipopolisaccharide (LPS, positive control). Prior to treatment of THP-1 cells, all MB-GF mixtures were sterilized by filtration through a 0.22 µm filter to remove potential contamination.

### 2.7 Nuclear extract and electrophoretic mobility shift assay (EMSA)

Preparation of nuclear extracts from THP-1 cells after their treatment and subsequent determination of NF-κB activity in the nuclear fractions of THP-1 cells by electrophoretic mobility shift assay (EMSA) was performed according to a previously described procedure (Siednienko et al., 2007). Briefly, THP-1 cells were washed with ice-cold PBS and disintegrated in ice-cold buffer A (10 mM HEPES pH 7.9, 10 mM KCl, 0.1 mM EDTA, 0.1 mM EGTA, 1 mM DTT, 1 mM PMSF, 0.1 mM Na3VO4, 0.1% NP-40) by gentle pipetting on ice for 15 min. After centrifugation at 12,000 × g for 1 min at 4°C, the supernatant from each sample was removed and the pellet was resuspended in three times the volume of ice-cold high-salt buffer B (20 mM HEPES, pH 7.9, 10 mM KCl, 1 mM EDTA, 1 mM EGTA, 420 mM NaCl, 20% glycerol, 1 mM DTT, 1 mM PMSF). The samples were then gently vortexed at 4°C for 30 min, centrifuged at 12,000 × g for 10 min at 4°C, the protein concentration in the supernatants was determined using the BCA protein assay, and the samples were then used for EMSA. To detect NF-κB complexes in nuclear extracts, the double-stranded oligonucleotide 5′ AGTGGGTGAGACTTTCCCAGGC-3′ (NF-κB probe, NF κB binding site underlined) was end-labeled with T4 polynucleotide kinase (Promega, Madison, WI, United States) in the presence of [γ-32P] ATP (Hartmann Analytic, Braunschweig, Germany). Nuclear extracts (10 μg of protein) were pre-incubated for 10 min at room temperature in reaction buffer (10 mM Tris–HCl pH 7.5, 50 mM NaCl, 1 mM MgCl2, 0.5 mM EDTA, 0.5 mM DTT, 4% glycerol and with 0.05 mg/mL Poly (dI-dC) used as a competitor for non-specific DNA binding proteins). Then, 70 fmol of 32P labeled NF-κB probe was added to the reaction mixtures and incubated for 30 min at room temperature. For EMSA, each sample was then electrophoresed on a 5% native polyacrylamide gel in TBE (×0.5). The gels were autoradiographed by exposure to storage phosphor screens for 24 h at 20°C, and DNA-bound NF-κB complexes were analyzed and quantified using Typhoon 8,600 Multi-Imaging System and Fragment Analysis software (Molecular Dynamics/Amersham Pharmacia Biotech, Sunnyvale, CA, United States).

## 3 Results

### 3.1 In silico determination of the MB susceptibility to glycation

**FIGURE 1:**
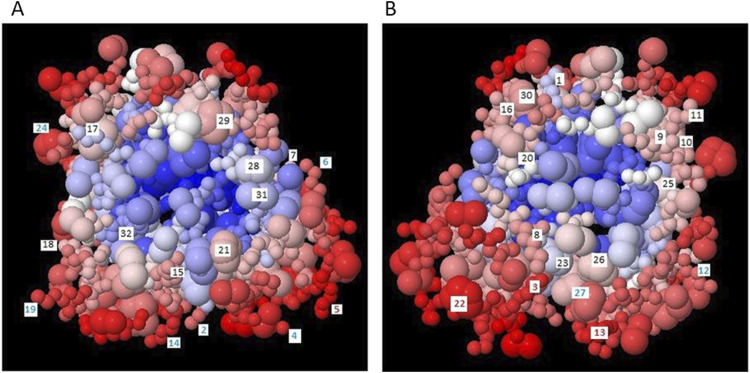
Spatial structure of myoglobin from horse heart muscle (PDB no.: 1NPF) detailing the residues that can undergo glycation: lysyl, arginyl and histidyl, determined using the BEpro Discontinuous B-cell Epitope Prediction (PEPITO) program according to Sweredoski and Baldi (2008); **(A)** view from the front side; **(B)** view after rotating 180°.

In order to obtain medium cross-linked AGEs, it was necessary to select a carrier protein with a low molecular weight and the presence of easily accessible lysyl, histidyl or arginyl residues (taking part in the Maillard reaction). We have selected myoglobin from horse heart muscle. This protein has a relatively low molecular weight (17 kDa), which makes it possible to obtain highly and medium cross-linked AGEs. Moreover, MB is easily glycated because the primary structure of this protein contains 19 lysyl, 2 argininyl and 11 histidyl residues, while there are no cysteinyl residues. Therefore, MB cannot undergo glycation by the Michael reaction, but only by the Maillard pathway. The spatial structure of myoglobin from horse heart muscle (PDB no.: 1NPF) was modeled in the BEpro Discontinuous B-cell Epitope Prediction (PEPITO) program (Sweredoski and Baldi, 2008) with the determination of amino acid residues that may undergo glycation (Figure 1). The amino acid residues that are most reactive to glycation factors are marked in red. These reactivities were determined based on the value of the epitope specificity coefficient E(r), resolved taking into account the environment surrounding a given amino acid residue and its spatial position in the protein structure. E(r) values for all lysyl, histidyl and arginyl residues are presented in Table 1. On their basis, it was found that five amino acid residues: Lys 45, Lys 50, Lys 96, Lys 98 and His 48 are particularly susceptible to glycation. In addition: Lys 42, Lys 47, Lys 56, Lys 87, Lys 147 and His 97 can also be glycated under conditions of increased concentration of the glycation factors. The remaining basic residues, which are not highly reactive based on the calculation of the E(r) value, can also be glycated due to the strong affinity of glycation factor to the hydrophilic groups present in these amino acids. Only His 24, Arg 31 and Arg 139, which have a negative E(r) value, are located inside the protein molecule and most likely cannot undergo glycation due to these spherical constraints.

**TABLE 1:** Values of the epitope specificity coefficient E(r) determined for each lysyl, histidyl and arginyl residue occurring in myoglobin from horse heart muscle (PDB no.: 1NPF).

No	Number of amin acid residue	E(r)
Lysyl		
1	Lys16	0.09
2	Lys42	0.9
3	Lys45	1.31
4	Lys47	1.24
5	Lys50	1.41
6	Lys56	0.90
7	Lys62	0.39
8	Lys63	0.67
9	Lys77	0.48
10	Lys78	0.59
11	Lys79	0.56
12	Lys87	0.90
13	Lys96	1.33
14	Lys98	1.24
15	Lys102	0.66
16	Lys118	0.59
17	Lys134	0.07
18	Lys145	0.52
19	Lys147	1.15
Histidyl		
20	His24	0.53
21	His36	0.53
22	His48	1.35
23	His64	0.18
24	His81	1.18
25	His82	0.32
26	His93	0.42
27	His97	0.92
28	His113	0.14
29	His116	0.58
30	His119	0.66
Arginyl		
31	Arg31	−0.05
32	Arg139	−0.16

### 3.2 Identification of AGEs generated on MB in HTMS and HTWS methods

**FIGURE 2:**
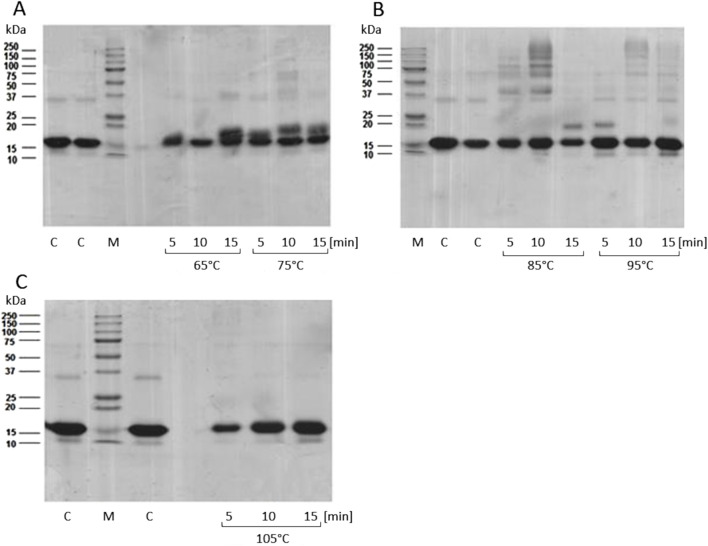
SDS-PAGE analysis of MB-mel products obtained by HTMS, with a MB/mel molar ratio of 1/100, carried out under various conditions of temperature (65°C, 75°C **(A)**, 85°C, 95°C **(B)** or 105°C **(C)**) and time (5, 10 or 15 min). 5 µg of sample was applied to the wells. C, control sample; MB/mel lyophilisates not exposed to microwaves; M, protein standard (Gen Ruler, BIO-RAD).

Syntheses were carried out for 6 different MB-GF mixtures: MB-ACR, MB-4HNE, MB-glc, MB-mel, MB-MGO and MB-T2N using HTMS and HTWS methods with different temperatures and reaction times. Then, each of the obtained products was subjected to SDS-PAGE to determine the optimal conditions to form medium cross-linked MB-AGEs. An example of such analysis for the MB-mel product obtained by HTMS method is shown in Figure 2. The most medium cross-linked MB-mel glycation products are formed during the synthesis carried out for 10 min at 85°C at a MB/mel molar ratio of 1/100. The use of a 100-fold excess of GF to MB in the HTMS method allowed obtaining soluble mixtures of MB-ACR, MB-glc, MB-mel and MB-T2N. Due to the preparation of insoluble products MB-4HNE and MB-MGO under the applied synthesis conditions, the molar ratios of MB/4-HNE and MB/MGO were reduced to 1/2 and 1/32, respectively. Additionally, due to the high reactivity of acrolein, reducing the molar ratio of MB/ACR to 1/32 allowed for even more efficient production of medium cross-linked, soluble AGEs. The HTWS synthesis of MB-GF products carried out under various time and temperature conditions led in each case to obtain soluble medium cross linked AGEs and large amounts of highly cross-linked, insoluble AGEs. To obtain soluble MB-4HNE products, the molar ratio of MB/4HNE was reduced to 1/32. Each sample was dark brown in color, the precipitate contained brown, insoluble aggregates. The brown color resulted from the formation of products in the Maillard reaction (caramelization reaction), which occurs between the carbonyl group of sugars and aldehydes and the amino group of protein side chains. Additionally, higher temperatures and the presence of another energy source (in our case, microwaves) intensify this reaction.

**FIGURE 3:**
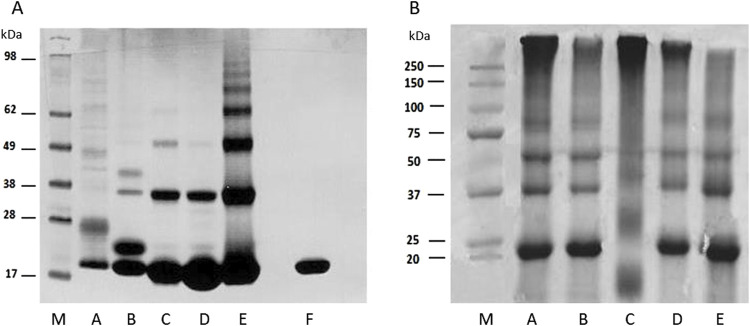
SDS-PAGE analysis of obtained glycation products. **(A)** Selected MB-GFs obtained by HTMS method; lanes: M–protein standard (Blue^®^ Plus2 Pre Stained Standard, Invitrogen, Waltham, MA, United States), A, MB-mel; B, MB-glc; C, MB-T2N; D, MB-4HNE; E, MB-ACR; F, unmodified MB (control). **(B)** Selected MB-GFs obtained by HTWS method; lanes: M, protein standard (Gen Ruler, BIO-RAD); A, MB-glc; B, MB-mel; C, MB-ACR; D, MB-T2N; E, MB-MGO. MB/GF molar ratios and synthesis conditions are presented in Table 2.

The optimal conditions for the MB-GF glycation using the HTMS and HTWS methods are presented in Table 2, and the obtained soluble MB-GF products are shown in Figure 3. The mixtures of glycation products obtained after HTMS synthesis are shown in Figure 3A. Glycation of MB by mel leads to the formation of low-cross-linked products with a molecular mass of 23.35 kDa, which is close to the molecular mass of MB (17.5 kDa), as well as medium-cross-linked products with molecular masses of 40.88 kDa, 48.15 kDa, 75.88 kDa and 151.19 kDa (Figure 3A, lane A). The MB-mel product with a mass of 23.35 kDa is the most abundant and constitutes approx. 41% of all products. However, all medium cross-linked MB-mel products account for as much as 58% of the mixture containing only soluble MB-mel glycation products. Glycation carried out by glucose leads to the formation of products with slightly different molecular masses: low-cross-linked products with a molecular mass of 19.52 kDa, close to that of myoglobin (17.5 kDa), and medium-cross-linked products with masses of 32.5 kDa, 39.03 kDa and 169.04 kDa (Figure 3A, lane B). Similar to the case of MB glycation by mel, the MB-glc product with a mass of 19.52 kDa is the most abundant (approx. 39%), and medium-cross-linked MB-glc products make up about 59% of all soluble products in the entire mixture. Glycation involving low-molecular-weight aldehydes such as T2N and 4HNE, results in medium-cross-linked products with the same molecular masses of 31.78 kDa and 52.3 kDa (Figure 3A, lane C and D). In the case of MB-T2N, a product with a mass of 145.65 kDa is also produced, while for MB-4HNE with a mass of 167.04 kDa. In the post-reaction mixture of MB-T2N, medium-cross-linked AGEs make up 35.3% of all products, while in the sample containing MB-4HNE products, medium-cross-linked AGEs constitute 24%. Glycation of MB with ACR leads to the formation of products with the following molecular masses: 32.14 kDa, 52.92 kDa, 73.17 kDa, 91.11 kDa and 106.88 kDa (Figure 3A, lane E). Medium-cross-linked MB-ACRs account for 52% of all soluble MB-ACR products. It is worth noting that in every type of MB-GFs obtained by the HTMS method, the low-cross-linked AGEs, whose molecular masses are close to that of MB, make up between 39% and 76% of all AGEs in a given sample. Glycation was most effective in the case of modification carried out by mel, as medium-cross-linked AGEs constitute 58% of all products in this sample. Moreover, the commercial MB sample used as a control did not show any glycation products (Figure 3, lane F).

**TABLE 2:** Synthesis conditions for obtaining soluble medium cross-linked advanced glycation end-products by HTMS and HTWS methods.

Type of MB/GF mixture	Temperature [^o^C]	Time [min]	molar ratio MB/GF (mol/mol)
HTMS			
MB-mel	85	10	1/100
MB-glc	95	10	1/100
MB-T2N	95	15	1/100
MB-4-HNE	75	10	1/2
MB-MGO	65	10	1/32
MB-ACR	75	10	1/32
HTWS			
MB-mel	95	120	1/100
MB-glc	95	120	1/100
MB-T2N	95	30	1/100
MB-4HNE	95	30	1/32
MB-MGO	75	30	1/100
MB-ACR	85	120	1/100

Gel analysis of samples after HTWS synthesis showed that during MB modification by mel, glc, ACR, T2N and MGO, both low- and medium-cross-linked glycation products are formed (Figure 3B). Glycation carried out by glc leads to the production of AGEs with molecular masses of 22.1 kDa, 34.79 kDa, 52.05 kDa, 89.12 kDa and 378.03 kDa, with low-cross-linked products (with a mass of 22.1 kDa) accounting for only 20% of the sample mass, and medium-cross-linked AGEs making up as much as 80% (Figure 3B, lane A). Moreover, medium-cross-linked AGEs obtained by HTWS method have significantly higher molecular masses (about 2 times) compared to MB-glc obtained by HTMS. Glycation carried out by mel results in products with the following molecular masses: 22.25 kDa, 35.75 kDa, 52.97 kDa, 52.97 kDa, 90.99 kDa and 311.39 kDa (Figure 3B, lane B). Low-cross-linked MB-mels (with a mass of 22.25 kDa) constitute 33% of the total sample, while medium-cross-linked products make up 67%. Glycation carried out by ACR leads to the formation of AGEs with molecular masses of: 19.26 kDa, 26.38 kDa, 42 kDa and 360.07 kDa (Figure 3B, lane C). Low-cross-linked MB-ACR products (with masses of: 19.26 and 26.38 kDa) constitute 26%, while medium-cross-linked MB-ACRs make up 74% of the sample. The applied HTWS conditions resulted in the production of mainly medium-cross-linked AGEs, with molecular mass 3-fold higher than MB-ACRs obtained using the HTMS method. Glycation of MB by T2N leads to the formation of products with molecular masses of 22.55 kDa, 35.76 kDa, 55.87 kDa, 96.92 kDa and 318.99 kDa (Figure 3B, lane D), with medium-cross-linked AGEs making up as much as 71% of the sample, while low-cross-linked MB-T2N products (with a mass of 22.55 kDa) account for 29% of the sample mass. Glycation of MB by MGO leads to the production of MB-MGOs with molecular masses of 21.95 kDa, 35.26 kDa, 53.91 kDa and 90.99 kDa (Figure 3B, lane E). The use of the HTWS method results in formation of low-cross-linked MB-MGOs (with a mass of 21.95 kDa), constituting 40% of the sample and 60% of medium-cross-linked AGEs.

### 3.3 Fluorescence properties of MB-GF products

**FIGURE 4:**
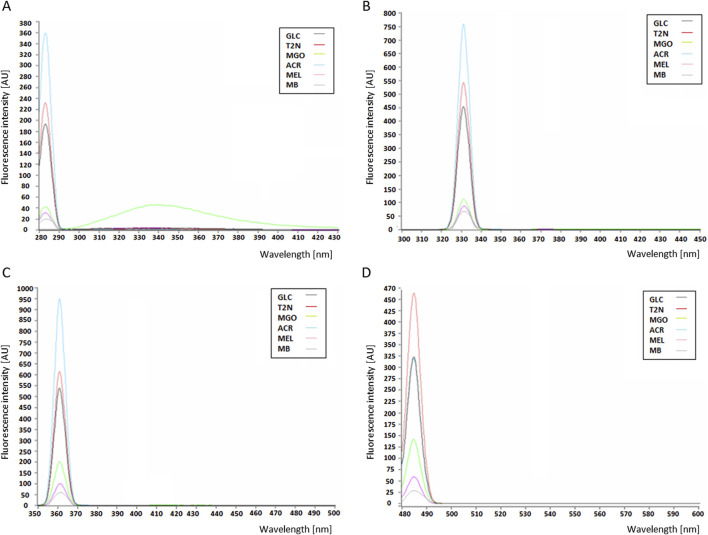
Profiles of fluorescence emission intensity of AGEs at a concentration of 0.05 mg/mL, upon excitation at a wavelength: **(A)** 280 nm; **(B)** 330 nm; **(C)** 360 nm; **(D)** 485 nm; with emission recorded in ranges: **(A)** 280–430 nm; **(B)** 300–450 nm; **(C)** 350–500 nm; **(D)** and 480–600 nm; MB, unmodified myoglobin; AGEs: MEL, MB-melibiose; ACR, MB-acrolein; MGO, MB-methylglyoxal; T2N, MB-trans-2-nonenal; GLC, MB-glucose.

The MB-GF mixtures obtained by HTWS and HTMS methods showed different fluorescent properties. When excited with 280 nm light, in the emission range of 280–430 nm, the fluorescence intensity increased in the following order: MB < MB-mel < MB-MGO < MB-glc < MB-T2N < MB-ACR (Figure 4A.). This order occurred regardless of the synthesis method, therefore Figure 4 shows the fluorescence intensity profiles only for AGEs obtained using the HTWS method. The same order of increase in the AGEs fluorescence intensity was observed upon excitation at 330 nm, with emission range of 300–450 nm (Figure 4B) and upon excitation at 360 nm, in the emission range of 350–500 nm (Figure 4C). This relationship also occurred regardless of the MB-GF synthesis method. However, when excited with light at 485 nm, and emission at 480–600 nm, the fluorescence intensity increased in a slightly different order: MB < MB-mel < MB-MGO < MB-glc ≤ MB-ACR < MB-T2N (Figure 4D).

After excitation of the tested samples with light at a wavelength of 280 nm, 330 nm or 360 nm, the MB-ACRs showed the highest fluorescence intensity compared to MB modified by glc, MGO, T2N. With the higher excitation wavelength in the range of 280–360 nm, a gradual increase in the signal emission intensity of these products was observed. Upon excitation at a wavelength of 485 nm, a decrease in the fluorescence intensity of AGEs was observed (Figure 4D). Under these conditions, MB-ACRs emitted a much weaker signal than MB-T2N products. The control containing unmodified MB emitted a very weak signal.

### 3.4 AGEs reactivity against anti-MAGE antibodies

**FIGURE 5:**
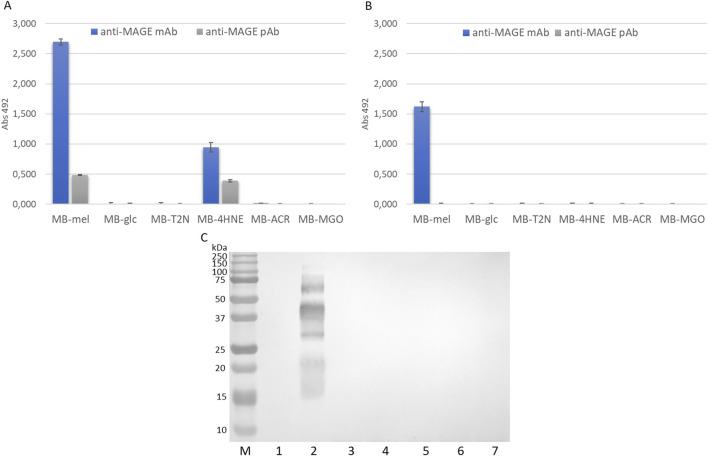
Reactivity of anti-MAGE antibodies against MB-GF glycation products. **(A)** ELISA of AGEs obtained by HTMS method; **(B)** ELISA of AGEs obtained by HTWS method; **(C)** Western blot analysis of AGEs obtained in HTMS with anti-MAGE monoclonal antibodies, M, protein marker; 1, unmodified MB; 2, MB-mel; 3, MB-glc; 4, MB-T2N; 5, MB-4HNE; 6, MB-ACR; 7, MB-MGO; mAb, monoclonal antibodies; pAb, polyclonal antibodies. The original blot is presented in Supplementary Figure S1.

In order to determine the reactivity of MB-GFs, obtained using the HTMS and HTWS methods, towards anti-MAGE monoclonal antibodies (Bronowicka-Szydełko et al., 2021; Gostomska-Pampuch et al., 2022a) and anti-MAGE polyclonal antibodies (Staniszewska et al., 2021), we performed ELISA and immunoblotting tests (Figure 5).

The results confirmed that MB-mel products obtained by HTMS (MAGEs) are the most reactive towards anti-MAGE antibodies among the tested AGEs (Figure 5A). A 5-fold higher reactivity of MB-mel towards monoclonal antibodies can be observed compared to polyclonal antibodies, which may indicate the high specificity of monoclonal antibodies. Moreover, MB-4HNE products obtained by HTMS also show reactivity towards anti-MAGE antibodies. In the case of monoclonal antibodies, the reactivity of MB-4HNE was 2.6-fold lower than that of MB-mel, and in the case of polyclonal antibodies, the reactivity of both AGEs was similar. The MB-mel product synthesized by HTWS reacts with anti-MAGE monoclonal antibodies, although the absorbance value is 1.7-fold lower than the absorbance value corresponding to MB-mel obtained by HTMS (Figure 5B). Additionally, MB-mel obtained by HTWS does not react with anti-MAGE polyclonal antibodies. The remaining MB-GF products are unrecognizable by any of the used antibodies. In order to qualitatively assess the reactivity of MB-GFs with anti-MAGE monoclonal antibodies, immunoblotting was performed (Figure 5C). The results indicate that only MB-mel HTMS products are reactive towards anti-MAGE monoclonal antibodies. The remaining AGEs obtained by HTMS and all MB-GFs obtained by HTWS did not show any reactivity towards anti-MAGE monoclonal antibodies.

### 3.5 Biological properties of obtained AGEs–activation of NF-κB transcription factor

**FIGURE 6:**
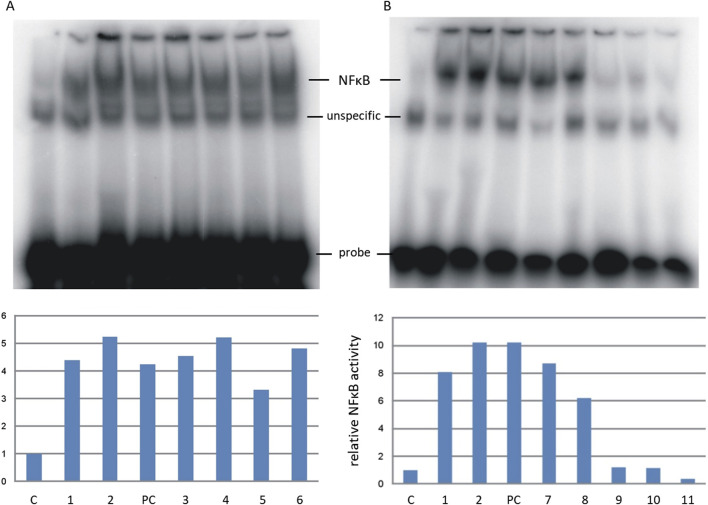
Relative activity of the NF-κB transcription factor in THP-1 cells treated with lipopolisaccharide (PC, positive control), unmodified myoglobin (1) and tested AGEs: MB-mel (2, 9), MB-ACR (3, 7), MB-4HNE (4), MB-glc (5, 8), MB-T2N (6, 11), MB-MGO (10). AGEs synthesized by the HTMS method are marked with numbers 2–6 **(A)**, and by the HTWS method by numbers 7–11 **(B)**. Bands corresponding to EMSA-determined NF-κB activity in nuclear extracts were quantified as described in Materials and Methods and expressed as fold NF-κB activity in untreated cells (C, negative control).

The aim of the research was to analyze the influence of protein glycation products on the activation of pro-inflammatory signaling molecules. Therefore, we analyzed the effect of AGEs on the activity of the NF-κB transcription factor measured by EMSA in human THP-1 monocytic cells. The experiment demonstrated the activation of NF-κB by MB-ACR, MB-4HNE, MB-mel, and MB-T2N obtained by HTMS (Figure 6A), whose relative NF-κB activity value was higher than the negative control, positive control and unmodified myoglobin, and was in the range: 4.5–5.2 activity units. This suggests that all these compounds have a pro-inflammatory effect, while the MB-glc product obtained by HTMS has a much lower value of relative NF-κB activity than unmodified myoglobin. The strongest activity was observed for MB-mel and MB-4HNE products, for which the relative activity was 5.2 units in relation to untreated cells. Experiments using mixtures of MB-GF products obtained by HTWS synthesis (Figure 6B) showed significant activation of NF-κB after treatment of cells with MB-ACRs and unmodified myoglobin, which caused an at least 8-fold increase in NF-κB activity in THP-1 cells. The MB-mel, MB-T2N and MB-MGO glycation products obtained by HTWS did not stimulate the activation of NF-κB, while the activation of this factor by MB-glc products was lower than that of the unmodified protein.

To sum up, glycation products obtained under HTWS conditions, apart from MB-ACR, do not activate the NF-κB factor in THP-1 cells, while those obtained under HTMS conditions, especially MB-mel and MB-4HNE, strongly induce the biosynthesis of this factor.

## 4 Discussion

Glycation products are created endogenously or exogenously. AGEs derived from food are well absorbed by the digestive system–studies conducted by Somoza et al. show that 30% of ingested CML is absorbed into the bloodstream (Somoza et al., 2005), so they are likely to lead to the same pathologies as glycation products formed *in vivo*. AGEs can also be formed by heating food in a microwave environment. Pilot studies have shown that heating infant formula in a microwave environment leads to the formation of various AGEs (Bronowicka-Szydełko et al., 2014). The amount of endogenous AGEs in the body depends on the concentration of reducing sugars, such as glucose or fructose, or reactive low-molecular-mass aldehydes derived from lipid oxidation, including 4-hydroxynonenal and trans-2-nonenal (Di Domenico et al., 2014). High concentrations of these compounds, occurring in numerous metabolic disorders such as diabetes, atherosclerosis, dyslipidemia, obesity and metabolic syndrome, lead to increased glycation of proteins, lipids, and nucleic acids (Yamagishi and Matsui, 2011). Consequently, micro- and macroangiopathies appear in the body, which, as they intensify, cause vascular changes (Gray and Jandeleit-Dahm, 2014) and may contribute to organ failure, including kidneys and heart (Foley et al., 2005).

MB has been used for years as a model to study protein glycation by various sugars, (Banerjee, 2017; Banerjee and Chakraborti, 2013; Banerjee et al., 2016; Bokiej et al., 2011; Liu et al., 2021). Nowadays, technology makes it possible to bioinformatically predict glycation sites in order to make hypotheses in a resource-saving way (time, materials, funds) on a preliminary basis. Such methods then require confrontation with *in vitro* methods. Examples of experimental studies of glycation sites on myoglobin include the work of Banerjee and co-workers, who showed that methylglyoxal can modify both Lys and Arg residues of MB, including Lys16, Lys42, Lys87, Lys133, Arg31 and Arg139 (Banerjee and Chakraborti, 2013; Banerjee et al., 2016). In a 2017 study, Banerjee describes a glyoxal-derived fluorescent AGE adduct of pentosidine between Lys-145 and Arg-139 MB residues (Banerjee, 2017). The result partially coincides with ours, although it should be remembered that in the cited work the model was myoglobin of human origin, while here it was horse myoglobin. Liu et al. (2021) reports that D-ribose undergoes rapid protein glycation of human myoglobin at lysine residues (Lys34, Lys87, Lys56, and Lys147), which is consistent for our modeling especially for the 56, 87 and 147 amino acid. We emphasize that *in silico* modeling of amino acid residues susceptible to glycation is an important tool in biomedical research, but only auxiliary to experimental methods, which enables quick and effective analysis of potential glycation sites and its impact on protein function. This modeling does not address the role of the glycation agent or the issue that the reaction conditions may be anhydrous. We intend to consider these issues in detail in a continuation of the project.

This study presents methods for obtaining AGEs in reactions involving physiological (4HNE, T2N, glc, MGO) or exogenous (ACR) glycation factors. The resulting products MB-4HNE and MB-T2N may serve as analogues of compounds formed during protein modification by aldehydes released during lipid peroxidation, while MB-glc and MB-MGO may represent those formed during hyperglycemia. Acrolein, on the other hand, is commonly considered a precursor to the formation of exogenous AGEs in the caramelization process during high-temperature food processing (Martins et al., 2000; Uribarri et al., 2003). It is worth noting that according to literature data, ACR is capable of glycation only through Michael addition, by breaking the disulfide bridges formed between cysteinyl residues. The obtained MB-ACR products using each of the methods (Figure 3) indicate the existence of a completely new, previously unknown pathway for obtaining this AGE. Furthermore, one hypothesis in contemporary glycobiology suggests that all amino acid residues, not just lysyl, arginyl, histidyl, and cysteinyl residues, may undergo glycation (Poulsen et al., 2013)–the obtained mixtures of MB-ACR (in each method) may support the validity of this hypothesis.

The most commonly used method for obtaining AGEs involves several weeks of incubation of a given glycation factor (usually glucose) with a model protein at a specific temperature in an aqueous environment or other polar solvent (Makita et al., 1992). Often, before the synthesis begins, the protein also needs to be incubated with H_2_O_2_ for several hours–oxidized protein is more susceptible to glycation (Vlassopoulos et al., 2013). The use of various conditions of time, temperature, pH, concentrations of glycation factors and model proteins [or model nucleotides such as GTP (Li et al., 2007)] allows for increased efficiency in obtaining AGEs. Moreover, some research groups have developed methods for obtaining AGEs in much shorter times, for example, Stanic-Vucinic et al. developed AGE synthesis in ultrasound, in an aqueous environment under non-denaturing conditions (Stanic-Vucinic et al., 2013). With its use, various products of β-lactoglobulin modification by glucose, galactose, fructose, lactose, ribose, and arabinose were obtained–the identification of obtained compounds was carried out using mass spectrometry, spectrophotometry, and fluorimetry. Additionally, Visentin et al. (2010) conducted AGEs synthesis (including pentosidine) in a microwave environment, also in aqueous conditions. Several hours of incubation of a model protein solution with a glycation factor under high-pressure conditions (850 MPa) can also lead to the formation of glycation products (Staniszewska et al., 2005). It is worth emphasizing that all of these synthesis methods take from several hours to several weeks and require the presence of a polar solvent as the reaction environment. This study presents a method for synthesizing various medium-crosslinked AGEs, involving conducting reactions under anhydrous conditions in a microwave environment (HTMS). In order to obtain medium-cross-linked AGEs, we have selected myoglobin from horse heart muscle as a carrier protein, due to its low molecular weight and the presence of easily accessible lysyl, histidyl or arginyl residues. The analysis of the epitope specificity coefficient E(r) values for all lysyl, histidyl and arginyl residues (Table 1) showed that only His 24, Arg 31 and Arg 139 most likely cannot undergo glycation due to these spherical constraints. As expected, in the work of Gostomska-Pampuch et al. (2022b), describing the mass spectrometry analysis of modifications occurring on myoglobin after reaction with melibiose, no glycation was shown on His 24 and Arg 31. However, the mel modification was identified on Arg 139 residue of MAGE product obtained in HTMS method. This indicates significant changes in the conformation of the MB molecule under glycation with this method. The reaction conditions for each MB/GF sample were optimized to obtain the highest amount of medium-cross-linked, soluble products–MB-ACR, MB-4HNE, MB-glc, MB-mel, MB-MGO, and MB-T2N–in the shortest possible time. Given that the used model protein–MB–contains 32 basic amino acid residues that can undergo glycation, the reactions were typically conducted with a molar ratio of MB/GF: 1/100, meaning statistically, there were 3 molecules of the given GF for each amino acid residue. This created a high probability of glycation for each of these residues. In the case of glycation of MB by reactive low-molecular-weight aldehydes: ACR and MGO, it was necessary to reduce the molar ratio of MB/GF to 1/32, and for the most reactive compound: 4HNE, which contains a hydroxyl group (crucial in the Maillard reaction) and a double bond (susceptible to Michael addition), the ratio was reduced to 1/2 (Table 2).

The optimalization of glycation conditions for MB by various GFs using HTMS and HTWS methods enabled the rapid and efficient production of 12 different MB-GF mixtures, containing water-soluble, medium-cross-linked AGEs (Figure 3). These mixtures were then subjected to experiments to determine some of the biological properties: reactivity with anti-MAGE antibodies, fluorescence ability, and activation of the nuclear factor NF-κB. Each experiment was performed at least three times. Knowing the fluorescence ability of obtained AGEs (using HTMS and HTWS methods) allowed for their classification according to the accepted nomenclature, where the ability to form cross-linking bonds and fluorescence properties are the determining factors. Fluorescence intensity analysis showed that the glycation products MB-ACR, MB-T2N, and MB-glc exhibit high fluorescence ability and most likely belong to the second group of AGE compounds (cross-linking compounds with fluorescent properties) (Figure 4). On the other hand, the glycation products MB-MGO and MB-mel exhibit weak fluorescence properties and most likely belong to the third group of AGE compounds (cross-linking compounds without fluorescent properties), regardless of the method used. The MB-ACR mixture exhibited the strongest fluorescence properties.

So far, several ELISA tests have been developed allowing for the detection of certain AGEs, with all serving to determine only those AGEs that have arisen in the process of protein modification by reducing sugars such as glucose (Takeuchi et al., 2001), fructose (Adisakwattana et al., 2014), or methylglyoxal (Rabbani et al., 2014). Some of them allow simultaneous determination of several AGEs, e.g., a mixture of CML/pentosidine. Often, the results of these tests suggest that the content of the examined AGEs in biological material is only slightly higher than in control material from healthy individuals (Aronson, 2003; Hudson et al., 2003; Haan, 2006; Humpert et al., 2006). Furthermore, so far, no tests have been developed in which the determined compounds would be products of protein modification by low-molecular-weight aldehydes derived from lipid peroxidation–a process occurring particularly intensively in insulin resistance, resulting from hyperglycemia (Mali et al., 2014).

The conducted ELISA and immunoblotting tests enabled the assessment of MB-GF reactivity with anti-MAGE antibodies, thereby determining the specificity of the tested antibodies (Figure 5). The immunoenzymatic studies also allowed for the detection of potential cross-reactivity, which often occurs with anti-AGE antibodies. For example, research conducted by various teams has shown that anti-CML monoclonal antibodies recognize not only CML but also CEL, indicating that these antibodies are not highly specific (Koito et al., 2004; Choi and Lim, 2009). The demonstration of anti-MAGE antibody reactivity exclusively with MB-mel products indicates that these antibodies are highly specific. However, the appearance of a reaction between MB-4HNE products obtained by the HTMS method and the tested antibodies could suggest that *in vivo* 4HNE leads to the formation of immunoreactive AGE10 (naturally occurring analogue to synthetic MAGE product), rather than melibiose, as the latter is not an endogenously occurring glycation factor [melibiose is produced during food fermentation by bacteria of the genus Bifidobacterium (O’Connell et al., 2013; O’Callaghan and van Sinderen, 2016), *Lactobacillus*, Lactococcus, Leuconostoc (Yoon and Hwang, 2008), and -yeast (Baú et al., 2015). It is also found in cocoa beans, honey, and processed soybeans (Schievano et al., 2017)]. This hypothesis, however, requires further confirmation in additional studies. Furthermore, the lack of reactivity of MB-glc and MB-MGO with anti-MAGE antibodies suggests that these antibodies do not recognize already known AGE epitopes such as CML and CEL, which are formed during glycation involving glucose (Matsumoto et al., 2009; Nagai et al., 2003; Nagai et al., 2008). Additionally, glucose is also a precursor of pentosidine, as confirmed by studies conducted by various research teams (Izuhara et al., 1999; Jono et al., 2002; Kato et al., 2001; Miyazaki et al., 2002). According to the literature, glycation of proteins by methylglyoxal results in the formation of CEL (Ahmed et al., 1997; Degenhardt et al., 1998), imidazolones (Jono et al., 2004), argpyrimidine (Oya et al., 1999), and so-called MOLD compounds (methylglyoxal-lysine dimer compounds with imidazole salt-type bonds) (Nagai et al., 2003; Nagaraj et al., 1996). The lack of reactivity of MB-MGO with the tested antibodies may indicate that these antibodies do not recognize CEL, imidazolones, argpyrimidine, or MOLD either. Therefore, the isolated anti-MAGE antibodies have a different specificity than those prepared by Oya et al. recognizing argpyrimidine (Oya et al., 1999) or by Konishi et al. (1994) targeting imidazolones.

It is noteworthy that the anti-MAGE antibody reaction is positive with MB-4HNE only in the ELISA method, the immunoblotting result is negative. This may be the result of differences in sensitivity and specificity of the methods. Typically, Western blot has lower sensitivity compared to ELISA, detecting analytes in the nanogram range. Western blot has the advantage of providing additional information about the molecular weight of the protein, for-which we do not discredit the immunoblotting result, but consider it as an auxiliary verification and confirmation of the undeniable anti-MAGE- MB-mel reaction. It is important to highlight that both immunoblotting and ELISA tests of MB-mel products obtained by the HTMS method demonstrated their analogous reactivity with anti-MAGE antibodies (Figure 5). In contrast, the reactivity of MB-mel obtained by the HTWS method was minimal. This suggest that the presence of water during a glycation reaction significantly reduces the efficiency of MAGE epitope formation, which is consistent with previously published results (Gostomska-Pampuch et al., 2022a; Staniszewska et al., 2021).

Numerous literature sources indicate the pro-inflammatory action of AGEs through their binding to the RAGE receptor (Zill et al., 2003). The AGE-RAGE complex activates the signaling pathway involving the NF-κB protein and enhances the expression of VCAM-1 proteins present in human umbilical vein endothelial cells (Kislinger et al., 1999; Koito et al., 2004). Studies conducted by Vlassara et al. (2002) demonstrated that patients on a diet containing a fivefold excess of CML exhibit significantly higher levels of pro-inflammatory cytokines. In this study, it was shown that the glycation of MB by ACR, glc, 4HNE, mel, MGO, and T2N using both HTMS and HTWS methods leads to the production of MB-GF products capable of activating the transcription factor NF-κB (Figure 6). The most strongly pro-inflammatory factors (highest relative NF-κB activity values) were found to be MB-mel and MB-4HNE products obtained by the HTMS method. Likewise, glycation products obtained by the HTWS method, which correspond to AGEs formed during food processing, also exhibited pro-inflammatory properties. The highest relative NF-κB activity was observed in cells treated with the MB-ACR mixture, which are glycation products commonly formed during caramelization and present in cigarette smoke (Martins et al., 2000; Uribarri et al., 2003). This is consistent with literature reports suggesting that long-term consumption of processed foods can lead to increased oxidative stress generated by various pro-inflammatory proteins, including NF-κB, which in turn stimulates insulin resistance and metabolic syndrome (Cai et al., 2008). Studies by Pötzsch et al. showed a link between an AGE-rich diet and NF-κB activation (Pötzsch et al., 2013). A practical conclusion follows, to sensitize the medical community to the prevention of insulin resistance and metabolic disorders by minimizing the consumption of advanced glycation products.

In conclusion, AGEs constitute a large group of compounds formed *in vivo* in random, non-enzymatic reactions of oxo-compounds with amino residues (of, most often, proteins), with different biological and physicochemical properties. Development of methods used in the *in vitro* synthesis and the assessment of the properties of compounds (representatives of AGEs produced *in vivo*) is crucial in striving to understand the pathomechanism of various clinical states, many of them being the plague of the XXIst century.

In the presented work, we have proved that the development of a synthesis method in anhydrous conditions with microwave (HTMS) allows to quickly acquire such AGEs that are created *in vivo*, which was confirmed by immunoenzymatic tests. Most likely, the microwave environment mimics oncotic blood pressure conditions. AGEs formed in HTMS had strong pro-inflammatory (activating NF-KB) and fluorescent properties. Moreover, glycation of MB with melibiose and 4-hydroxynonenal allows obtaining products recognized by the same novel anti-AGE10 antibodies developed by our team.

## Data Availability

The raw data supporting the conclusions of this article will be made available by the authors, without undue reservation.
