# Supplementary material for: Anhydrous microwave synthesis as efficient method for obtaining model advanced glycation end-products
Source: Front Mol Biosci. 2024 Nov 1;11:1484196. doi: 10.3389/fmolb.2024.1484196 (PMC11599739; doi:10.3389/fmolb.2024.1484196)
Supplement: Supplementary file 1 [file Image1.pdf]

Supplementary Material

1 Supplementary Figures

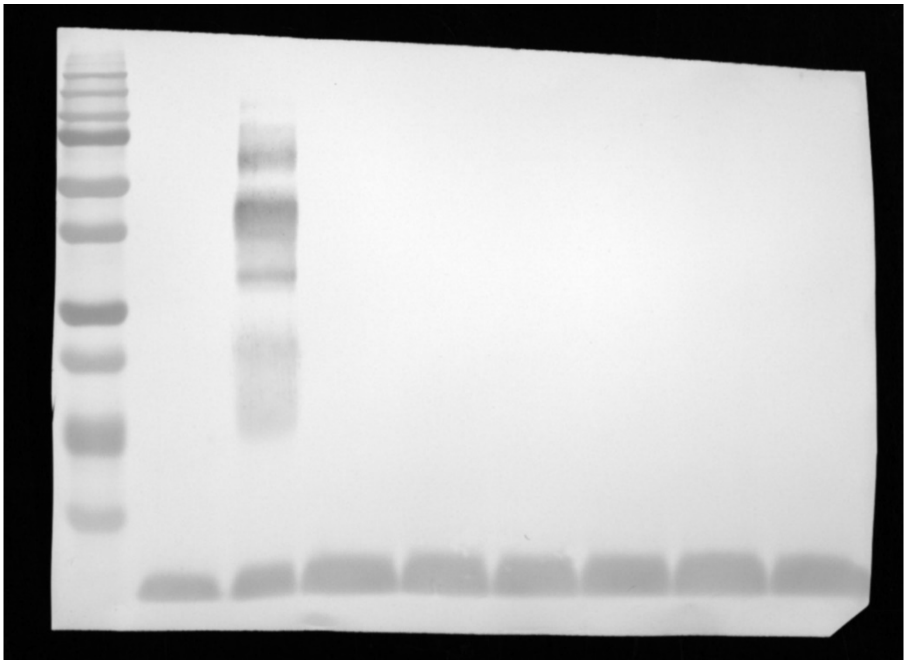

Supplementary Figure 1. Uncropped blot of Figure 5C in main text of manuscript. Western blot analysis of AGEs obtained in HTMS with anti-MAGE monoclonal antibodies.
